# Poisoning by Oil of Tansy

**Published:** 1852-08

**Authors:** 


*Poisoning by Oil of Tansy.* By W. W. Ely, M. D., of Rochester, N. Y.

The subject of the following painful occurrence, was a respectable young lady, in ordinary health, engaged at the time in teaching school. Having arrived at her menstrual period, she procured what she supposed was the essence of tansy, designing to take it to promote the catamenial discharge. On the evening of August 15, 1836, she took *one teaspoonful* of the medicine, which proved to be *oil of tansy.* From the speedy supervention of alarming symptoms a messenger was sent for me, a distance of two miles. Being unable to attend personally she was promptly visited by my partner. The oil, however, had operated so energetically and rapidly that on his arrival nothing seemed likely to be of any avail, and nothing of any consequence was done.

From the record which I made at the time, it appears that she first complained of dizziness and became insensible in about ten minutes—a succession of convulsions supervened, with frothing at the mouth, laborious respiration and irregular pulse, and she died in *one hour and a quarter* after taking the oil.

It may be proper to add that another young lady in the family, also took of the medicine at the same time, but vomited very soon, and sufferéd no inconvenience.

